# WalnutGDB: an integrated genomic portal for advancing Juglandaceae research and breeding

**DOI:** 10.1093/hr/uhag173

**Published:** 2026-04-28

**Authors:** Honghua Zhang, Langqin Luo, Songyang Li, Zhenyang Shua, Wen Yao, Xiang Luo, Haifang Hu, Xin Chen, Shaowen Quan, Yongqiang Chen, Zhongzhong Guo, Rui Zhang

**Affiliations:** Xinjiang Production and Construction Corps Southern Xinjiang Characteristic Agroforestry Technology Innovation Center, Tarim University, Alar 843300, China; The National and Local Joint Engineering Laboratory of High Efficiency and Superior-Quality Cultivation and Fruit Deep Processing Technology of Characteristic Fruit Trees in South Xinjiang, Tarim University, Alar 843300, China; College of Life Science and Technology, Tarim University, Alar 843300, China; Xinjiang Production and Construction Corps Southern Xinjiang Characteristic Agroforestry Technology Innovation Center, Tarim University, Alar 843300, China; The National and Local Joint Engineering Laboratory of High Efficiency and Superior-Quality Cultivation and Fruit Deep Processing Technology of Characteristic Fruit Trees in South Xinjiang, Tarim University, Alar 843300, China; College of Life Science and Technology, Tarim University, Alar 843300, China; College of Life Sciences, Henan Agricultural University, Zhengzhou 450046, China; College of Life Sciences, Henan Agricultural University, Zhengzhou 450046, China; College of Life Sciences, Henan Agricultural University, Zhengzhou 450046, China; Institute of Fruit and Vegetable, Xinjiang Academy of Agricultural Sciences, Urumqi 830091, China; Xinjiang Academy of Forestry Sciences, Urumgi 830092, China; Walnut and Chestnut Nursery in Tai’an National Fruit Germplasm, Shandong Institute of Pomology, Tai’an, Shandong 271000, China; College of Agriculture, Shihezi University, Shihezi, Xinjiang 832003, China; Xinjiang Production and Construction Corps Southern Xinjiang Characteristic Agroforestry Technology Innovation Center, Tarim University, Alar 843300, China; The National and Local Joint Engineering Laboratory of High Efficiency and Superior-Quality Cultivation and Fruit Deep Processing Technology of Characteristic Fruit Trees in South Xinjiang, Tarim University, Alar 843300, China; Xinjiang Production and Construction Corps Southern Xinjiang Characteristic Agroforestry Technology Innovation Center, Tarim University, Alar 843300, China; The National and Local Joint Engineering Laboratory of High Efficiency and Superior-Quality Cultivation and Fruit Deep Processing Technology of Characteristic Fruit Trees in South Xinjiang, Tarim University, Alar 843300, China; Xinjiang Production and Construction Corps Southern Xinjiang Characteristic Agroforestry Technology Innovation Center, Tarim University, Alar 843300, China; The National and Local Joint Engineering Laboratory of High Efficiency and Superior-Quality Cultivation and Fruit Deep Processing Technology of Characteristic Fruit Trees in South Xinjiang, Tarim University, Alar 843300, China

Dear Editor,

The Juglandaceae family comprises approximately 8 genera and 63 species. Members of this family have been cultivated for centuries owing to their nutrient-rich edible nuts and high-quality timber, conferring considerable economic and ecological importance worldwide [1].

Recent years have witnessed rapid expansion of genomic research in Juglandaceae, particularly in the key genera *Juglans* and *Carya* [1–4]. Advances in high-throughput sequencing technologies have enabled comprehensive genome assembly and systematic analysis across these taxa, yielding valuable insights into the genetic basis of agronomically important traits, including fruit quality, disease resistance, and stress tolerance, as well as ecological adaptation and resilience to climate change [3, 4].

However, despite the growing availability of Juglandaceae genomic and multi-omics data, these resources remain scattered across disparate repositories, creating significant barriers to efficient data management and integrative analysis [5]. To address this gap, we developed WalnutGDB, a centralized genomic database for the Juglandaceae family (Fig. 1). WalnutGDB integrates 21 genome assemblies representing 7 genera and 18 species, together with diverse multi-omics resources, including 10 211 760 single-nucleotide polymorphisms (SNPs) derived from 815 walnut accessions [4], structural variants (SVs) identified across multiple walnut genomes, pan-genomic frameworks, and eight gene expression datasets spanning five species (Fig. 1A). Furthermore, WalnutGDB incorporates a suite of analytical tools, including BLAST, JBrowse, primer design, and GO/KEGG enrichment analysis, enabling efficient data exploration and facilitating in-depth investigation of economically and ecologically important traits across Juglandaceae.

**Figure 1:**
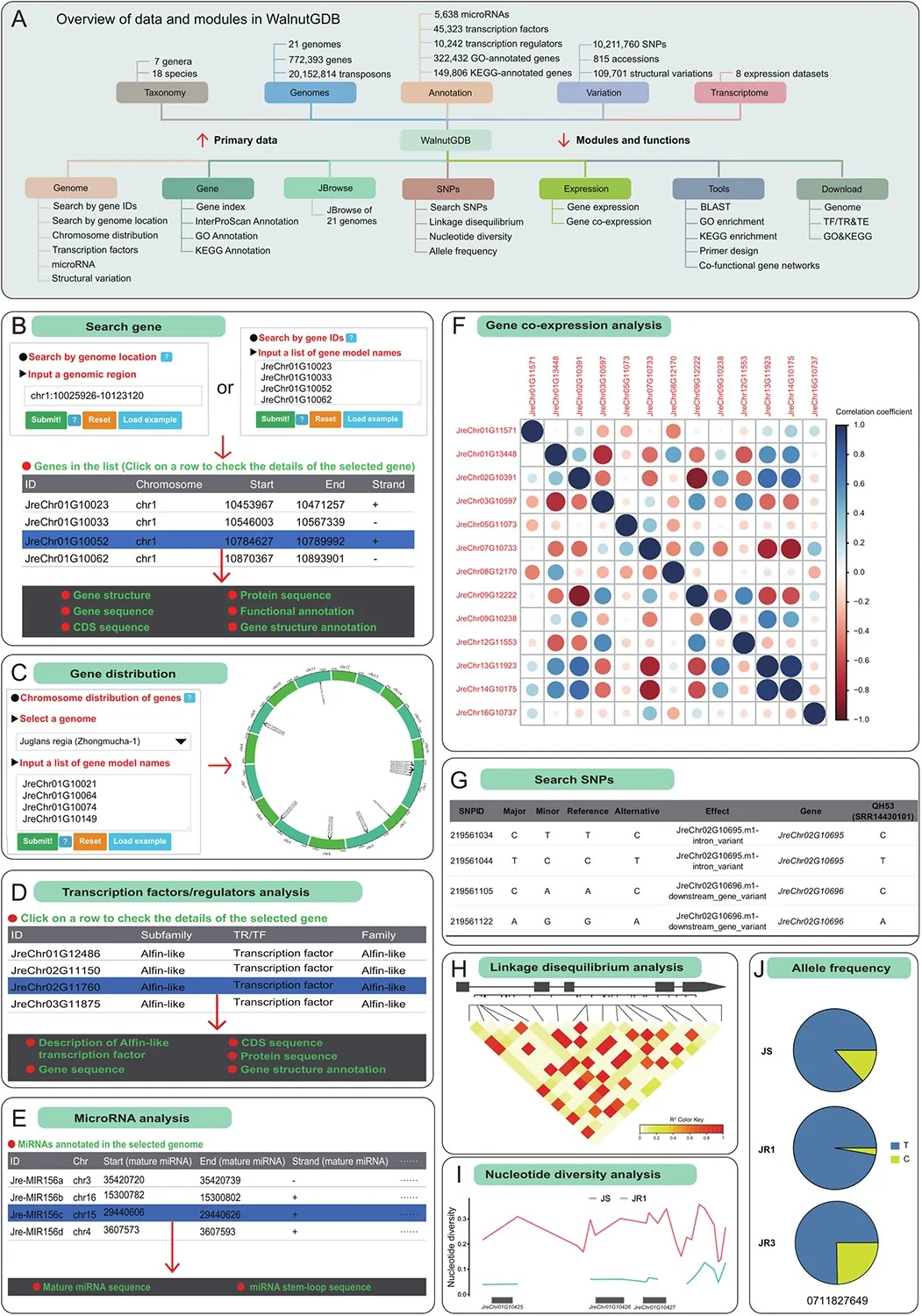
Overview of WalnutGDB architecture and functional modules. (A) Schematic overview of the data content and functional modules in WalnutGDB. (B) The ‘Genomes’ module supports retrieval by genomic region or gene IDs, providing access to gene structure, CDS, protein, and functional annotation. (C) Circular visualization of chromosomal gene distributions for selected genomes. (D) The ‘Transcription factors/regulators analysis’ sub-module displays subfamily and family classifications with links to detailed annotations. (E) The ‘MicroRNA analysis’ sub-module provides positional information and access to mature and stem-loop sequences. (F) The ‘Gene co-expression analysis’ function displays pairwise correlation coefficients as an interactive matrix. (G) The ‘Search SNPs’ function returns genotype information including position, alleles, functional effects, and associated gene IDs. (H) Linkage disequilibrium patterns visualized as a heatmap. (I) Nucleotide diversity across genomic regions displayed as sliding-window line charts. (J) Allele frequency distributions presented as pie charts.

WalnutGDB was constructed by systematically collecting publicly available datasets from NCBI, NGDC, Figshare, GigaDB, and The Genome Data of *Juglans* [1], encompassing genome assemblies, gene annotations, and transcriptomic datasets (Fig. 1, Fig. S1). CDS and protein sequences were directly extracted and standardized from these resources. Pan-genomic frameworks were independently constructed, and miRNAs were identified using a unified pipeline. The collected SNP datasets were subjected to rigorous quality control and functional annotation, and SVs were identified from the collected genome assemblies.

WalnutGDB currently houses 21 genome assemblies from 18 species across seven genera of Juglandaceae, with gene annotations sourced from the original publications documenting 772 393 genes in total (Fig. S2). Transposable elements were uniformly annotated using a standardized pipeline and unified repeat library, yielding 20 152 814 transposons. GO terms were assigned to 322 432 genes and KEGG pathways to 149 806 genes, and 45 323 transcription factors along with 10 242 transcriptional regulators were identified. The quality-controlled SNP dataset comprises 10 211 760 high-quality loci, and structural variation data from multiple walnut genomes were likewise integrated, together with eight gene expression datasets retrieved from public repositories.

WalnutGDB was developed using the R/Shiny framework and delivers multi-omics integration and visualization through a series of functional modules with user-friendly graphical interfaces. The platform supports efficient data retrieval, browsing, downloading, and analysis, serving as a valuable resource for Juglandaceae genomics research and genetic improvement. The database is organized into several core functional modules, including Species, Genomes, Genes, Expression, SNPs, JBrowse, and Tools, to facilitate comprehensive multi-omics exploration (Fig. S3).

The ‘Species’ module provides a centralized information hub for 18 Juglandaceae species encompassing 21 genome assemblies. For each species, users can access botanical descriptions, representative images, and key assembly statistics, including total assembly length, scaffold count, and scaffold N50, along with bibliographic references linking directly to the original data sources.

The ‘Genomes’ module integrates six major functions: gene ID search, genome location search, chromosomal gene distribution, transcription factor/regulator analysis, microRNA analysis, and structural variation analysis (Fig. 1B). All 772 393 gene records are searchable by gene ID or genomic region, with results providing genomic coordinates, structural and functional annotations, and corresponding sequences; region-based queries additionally display transposable elements. WalnutGDB also offers circular visualization of chromosomal gene distributions (Fig. 1C). Dedicated sub-modules further enable users to retrieve detailed structural annotations and CDS/protein sequences for transcription factors and regulators across 20 genomes (Fig. 1D), extract mature and precursor sequences alongside positional information for microRNAs across 21 genomes (Fig. 1E), and explore chromosomal rearrangements through structural variation queries in *Juglans*.

WalnutGDB integrates the high-performance genome browser JBrowse to support comprehensive visualization and exploration of 20 annotated genomes (Fig. S4). Users can inspect genome sequences, gene annotations, GO/KEGG classifications, transcription factors/regulators, and transposable elements across multiple genome tracks. Specialized SNP tracks are additionally provided for the *Juglans regia* cv. Zhongmucha-1 genome [1], facilitating investigation of SNP effects on gene function and genetic diversity across the 815 accessions.

The ‘Expression’ module enables users to retrieve gene expression levels and export results for downstream analysis. The module further supports heatmap visualization of expression patterns across tissues or samples and a co-expression analysis framework for exploring coordinated expression dynamics under diverse conditions, facilitating the elucidation of potential functional relationships and regulatory networks (Fig. 1F).

Leveraging the eggNOG database, WalnutGDB delivers comprehensive functional annotations for protein-coding genes across 20 annotated Juglandaceae genomes, encompassing 239 340 GO terms (assigned to 322 432 genes) and 2773 KEGG pathways (assigned to 149 806 genes). The platform further enables users to perform GO/KEGG annotation and enrichment analyses on user-submitted gene sets, with results dynamically visualized as bar charts or scatter plots and readily available for download for subsequent analysis (Fig. S4).

For genetic variation analysis, WalnutGDB provides four interactive tools for SNP search, linkage disequilibrium analysis, nucleotide diversity analysis, and allele frequency analysis (Fig. 1G–J). Users can query SNP genotypes, positions, alleles, and predicted functional effects within specified genes or genomic regions, and visualize the results through heatmaps, diversity plots, and allele frequency charts.

In addition, WalnutGDB integrates a BLAST module for homology searches across 20 high-quality Juglandaceae genomes and a Primer Design module for designing PCR primers targeting the identified SNP loci (Fig. S4). The database also offers open access to downloadable resources, including genome assemblies, CDS, protein sequences, and functional annotations.

In summary, WalnutGDB provides a centralized and comprehensive genomic resource for the Juglandaceae family. By integrating multi-omics data across seven genera and 18 species, the database effectively resolves long-standing data fragmentation and offers a unified platform for comparative and functional genomics research. The integration of pan-genomic frameworks with structural variation and SNP datasets enables comprehensive investigation of genetic diversity, chromosomal rearrangements, and their roles in species differentiation and phenotypic variation. In future updates, we will continue to incorporate additional multi-omics datasets and phenotypic information to support GWAS and provide long-term data resources for both fundamental research and molecular breeding in Juglandaceae.

## Data Availability

All data integrated within WalnutGDB are freely accessible at https://walnut.ac.cn/WalnutGDB/. Supplementary methods and datasets supporting this article have been deposited in the Figshare repository (https://doi.org/10.6084/m9.figshare.31834975).
